# Expression of EGFR in Pituitary Corticotroph Adenomas and Its Relationship With Tumor Behavior

**DOI:** 10.3389/fendo.2019.00785

**Published:** 2019-11-14

**Authors:** Xiaohai Liu, Ming Feng, Congxin Dai, Xinjie Bao, Kan Deng, Yong Yao, Renzhi Wang

**Affiliations:** ^1^Department of Neurosurgery, Xuanwu Hospital Capital Medical University, Beijing, China; ^2^Department of Neurosurgery, Peking Union Medical College Hospital, Chinese Academy of Medical Sciences and Peking Union Medical College, Beijing, China

**Keywords:** pituitary corticotroph adenoma, Cushing disease (CD), epidermal growth factor receptor (EGFR), tumor recurrence, EGFR pathway

## Abstract

**Objective:** Epidermal growth factor receptor (EGFR) has been found to localize in several human neoplasms and has been shown to have a significant correlation with adenomaigenesis and patient prognosis. EGFR is also overexpressed in pituitary corticotroph adenomas. However, its clinical significance and relationship with tumor behavior, especially tumor recurrence status, remain obscure. The purpose of the present study was to identify the expression patterns of EGFR and its downstream signaling pathway molecules in pituitary corticotroph adenomas and to investigate the association of EGFR with clinicopathological characteristics and tumor recurrence.

**Methods:** Fifty-two sporadic pituitary adenoma specimens and six normal pituitary glands were collected. The expression levels of EGFR and its downstream signaling molecules in each sample were evaluated and quantified using immunohistochemistry and Western blot. The relationships of EGFR expression with clinicopathological characteristics and tumor recurrence status were analyzed.

**Results:** EGFR was overexpressed in 55.8% of the pituitary corticotroph adenomas and in 1 of 6 of the normal adenohypophysial tissues. The expression degree was significantly higher in pituitary corticotroph adenomas than in normal adenohypophysial tissues. In EGFR-overexpressing adenomas, the downstream pathway phosphorylated Erk (p-Erk) was also significantly activated. Moreover, the expression levels of EGFR were positively correlated with the adrenocorticotropic hormone (ACTH) and cortisol levels but were not correlated with age, sex or symptom duration. The expression levels of EGFR, phosphorylated EGFR (p-EGFR) and p-Erk were significantly up-regulated in the recurrent adenoma group compared with those in the non-recurrent adenoma group (all *p* < 0.05). The expression levels of EGFR were strongly correlated with the recurrence-free interval (*p* = 0.005, CC = −0.31).

**Conclusion:** The expression levels of EGFR and its downstream pathway components were significantly increased in pituitary corticotroph adenomas compared to the levels in normal adenohypophysial tissues. EGFR expression levels were positively associated with the ACTH and cortisol levels and with tumor recurrence status. Pituitary corticotroph adenomas with high EGFR expression levels were correlated with an increased recurrence rate and a decreased recurrence-free interval. EGFR could be used as a promising biomarker for predicting pituitary corticotroph tumor recurrence.

## Introduction

Pituitary corticotroph adenomas arising from pituitary corticotroph cells account for 70% of endogenous Cushing disease (CD) cases (1, 2). CD is associated with increased morbidity and mortality mainly due to metabolic and cardiovascular complications, osteoporosis, psychiatric changes, and cognitive impairment as a result of the excessive production of adrenocorticotropic hormone (ACTH), which induces adrenal hypercortisolemia (3). The primary treatment is transsphenoidal surgical excision of the adenoma, and experienced pituitary surgeons can achieve initial remissions rate ranging from 65 to 90% for microadenomas and <65% for macroadenomas (4). However, curative surgery is challenging, as postoperative recurrence rates at 10 years are as high as 12–45%, even though most CD patients (90%) have adenomas smaller than 1 centimeters in diameter (4). For those recurrent adenomas, medical therapy to inhibit adrenal function, radiotherapy, and bilateral adrenalectomy has been used with limited efficacy (5). Therefore, early prediction of tumor recurrence is of great importance.

Epidermal growth factor receptor (EGFR) is expressed to varying degrees in human anterior pituitary glands, non-functional and functional pituitary adenomas, including corticotroph adenomas (6). Moreover, EGFR can regulate the transcription of proopiomelanocortin (POMC), which is a precursor of ACTH, indicating its role in the pathogenesis of pituitary corticotroph adenomas (7). The function of EGFR in the adenomaigenesis of CD has been highlighted by the finding of ubiquitin specific peptidase 8 (USP8) mutations in 35–62% of corticotroph adenomas (8, 9). The USP8 mutation (14-3-3 somatic mutations) induces corticotroph EGFR adenoma signaling by rescuing EGFR from lysosomal degradation and enhancing EGFR accumulation (9). However, the clinical significance of EGFR and its relationship to adenoma recurrence in corticotroph adenomas are still obscure. In the present study, we identified the expression patterns of EGFR and its downstream pathway in pituitary corticotroph adenomas and investigated its association with clinicopathological characteristics and tumor recurrence. Using this method, we hope to identify the clinical significance of EGFR, especially in predicting corticotroph adenoma recurrence.

## Materials and Methods

### Patients and Samples

Between Jan. 2012 and Dec. 2014, two hundred and eleven patients with CD received surgery at Peking Union Medical College Hospital (PUMCH). All operations were performed by Dr. Renzhi Wang. This retrospective study was reviewed and approved by the Research Ethics Committee of PUMCH. All patients signed written informed consent forms. All patients were diagnosed as CD and received no medicine or radiotherapy before their operation. All the patients included in this studies were micro-tumor with Knosp grade 1–2 and had immediate remission after operation and no residual tumor left after surgery in the MR, and the levels of cortisol, ACTH, or UFC levels after surgery were normal. All patients were persuaded to be reassessed periodically at 1, 3, 6, and 12 months after the surgery and yearly thereafter. A pituitary MRI was performed at 3, 6, 12 months and once a year after the surgery. As the late-night salivary cortisol test or the desmopressin stimulation test were not available in our hospital, the diagnostic criteria of the recurrence of CD were an insufficient suppression of serum cortisol after an overnight dose of 1 mg dexamethasone and elevated 24 h urinary cortisol levels associated with a reappearance of cushingoid symptoms caused by hypercortisolism after resection which may overlook patients with earlier recurrence. The assay (ADVIA Centaur) used to measure serum cortisol was purchased from Siemens Healthcare Diagnositic Inc. with a normal range from 5.27 to 22.45 μg/dL at 8 am. The 24 h UFC was measured by the same assay with a normal range from 20.9 to 292.3 μg/24 h. After an intensive follow-up (mean 44.8 months, range 37–69 months), 22 patients had tumor recurrence as defined above. There were 4 men, and 18 women with a mean age of 38.3 years and a range of 18–61 years. The patients in the non-recurrent group (*n* = 30) were selected which were age- and sex-matched with the patients in the recurrent group. The six normal pituitary glands were obtained from donors who died of non-neurological diseases in the Department of Anatomy at PUMC, including 3 male and 3 female donors aged 35 to 78 years.

### Immunohistochemical Staining

Tissue specimens were fixed in 10% formalin and embedded in paraffin, and five-micrometer sections were cut from the paraffin blocks. Tissues were stained using anti-EGFR (Cell Signaling Technology, Boston, MA). The sections incubated with phosphate-buffered saline alone were used as negative controls. To calculate the integrated optical density (IOD) value, Image-Pro Plus 6.0 software (Media Cybernetics, Inc., Silver Spring, MD, USA) was used for three randomly selected fields of view (200×). A semiquantitative assessment of the immunohistochemical reactions of EGFR was used to score the staining as 0 (negative, IOD < 0.1), 1+ (low, 0.1–0.4), 2+ (intermediate, 0.4–0.6), 3+ (high, 0.6–0.8), or 4+ (very high, >0.8). Then, immunohistochemical protein expression was scored blindly by two pathologists using a conventional optical microscope (Olympus, Tokyo, Japan).

### Western Blotting

Expression of EGFR and its signal transduction molecules, namely, phosphorylated EGFR (p-EGFR), total Akt, phosphorylated Akt (p-Akt), total Erk, and phosphorylated Erk (p-Erk), were analyzed using Western blotting. Briefly, cell lysates were prepared in the adenoma and normal pituitary gland tissues in 100 μL of radioimmunoprecipitation assay buffer (Sigma-Aldrich) containing protease inhibitor (Roche Molecular Biochemicals) and phosphatase inhibitor cocktails (Sigma-Aldrich). Then, the protein concentrations of the lysates were determined using bicinchoninic acid protein assay reagent (Thermo Scientific). Cell lysates were separated on 4–12% NuPAGE Bis-Tris gels and transferred onto a polyvinylidene fluoride membrane (Invitrogen) and probed using standard techniques with primary antibodies against anti-EGFR antibody, p-EGFR), total Akt, p-Akt, total extracellularly regulated kinase (Erk) and p-Erk (all purchased from Technology, Boston, MA). A peroxidase-conjugated secondary antibody (Amersham ECL HRP-Linked; Life Sciences) was incubated with the membrane for 1 h. The intensity of the antibody binding proteins was detected and calculated by a chemiluminescence detection system (Amersham Biosciences).

### Statistical Analyses

All statistical analyses were performed using SPSS 17.0 software (SPSS Inc., Chicago, IL, USA). The data are expressed as the means ± SD, with *p* < 0.05 considered statistically significant. The differences in EGFR expression among groups were compared using independent-samples *t*-test. The relationship between EGFR and clinicopathological characteristics was evaluated using the Mann-Whitney test.

## Results

### Expression and Location of EGFR in Pituitary Corticotroph Adenomas

**Figure 1:**
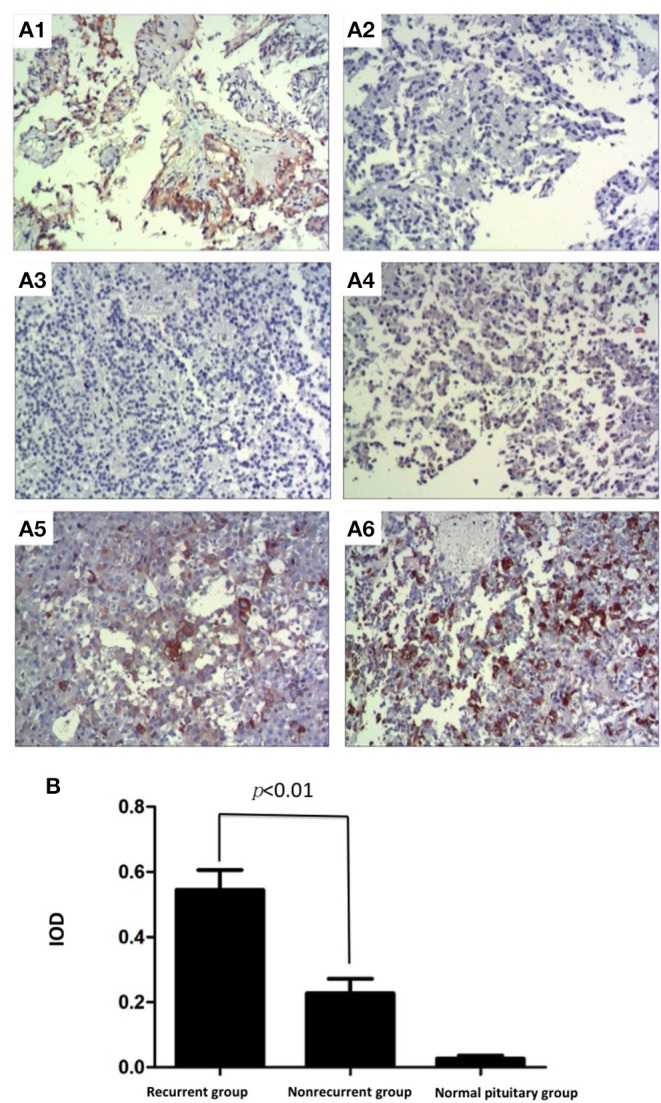
Expression and location of EGFR in pituitary corticotroph adenomas and normal pituitary glands. **(A1)** Strong immunoreactivity of EGFR was detected on the cellular membrane and in the cytoplasm of thyroid papillary carcinoma, IOD: 0.722 (200 × magnification). **(A2)** Thyroid papillary carcinoma did not show any immunoreactivity when the primary antibody directed against EGFR was replaced with phosphate-buffered saline (200 × magnification). **(A3)** No immunoreactivity of EGFR was detected in a normal pituitary gland, IOD: 0.012 (200 × magnification). **(A4)** Non-recurrent pituitary corticotroph adenomas, IOD: 0.190 (200 × magnification). (200 × magnification). **(A5)** Recurrent pituitary corticotroph adenomas, IOD: 0.406 (200 × magnification). **(A6)** Recurrent pituitary corticotroph adenomas, IOD: 0.640 (200 × magnification). **(B)** Mean EGFR IOD values in recurrent and non-recurrent pituitary corticotroph adenomas and in normal pituitary glands. Values are expressed as the means ± SDs. Compared with normal pituitary glands, pituitary glands with both types of pituitary corticotroph adenomas had significantly increased mean EGFR IOD values (both *p* < 0.001). Compared with the non-recurrent group, the recurrent group had significantly increased mean EGFR IOD values (*p* < 0.01).

To verify the expression and location of EGFR in pituitary corticotroph adenomas and normal pituitary glands, IHC staining was used and showed strong membrane and cytoplasmic EGFR immunoreactivity in 29 of 52 (55.8%) pituitary corticotroph adenomas and in 1 of 6 (16.6%) normal pituitary glands. Representative images of IHC staining in pituitary corticotroph adenomas and normal pituitary glands are shown in Figure 1A. There were 14 EGFR-positive adenomas in 20 (70%) recurrent adenomas and 15 EGFR-positive adenomas in 32 (46.9%) non-recurrent adenomas. In the 20 recurrent corticotroph adenomas, the degree of immunostaining was 4+ in 4 cases, 3+ in 4 cases, 2+ in 4 cases, and 1+ in 2 cases, while the degree of immunostaining was 4+ in 1 case, 3+ in 2 cases, 2+ in 7 cases, and 1+ in 6 cases in the 32 non-recurrent adenomas. There was only 1+ in 1 case in the 6 normal pituitary glands. The mean IODs for recurrent corticotroph adenomas, non-recurrent corticotroph adenomas and normal pituitary glands were 0.576, 0.216, and 0.016, respectively. Compared with normal pituitary glands, both recurrent and non-recurrent pituitary corticotroph adenomas had significantly increased mean EGFR IOD values (both *p* < 0.001). Compared with the non-recurrent group, the recurrent group had significantly increased mean EGFR IOD values (*p* < 0.01, Figure 1B).

### Expression of EGFR and Its Downstream Pathways in Pituitary Adenomas

**Figure 2:**
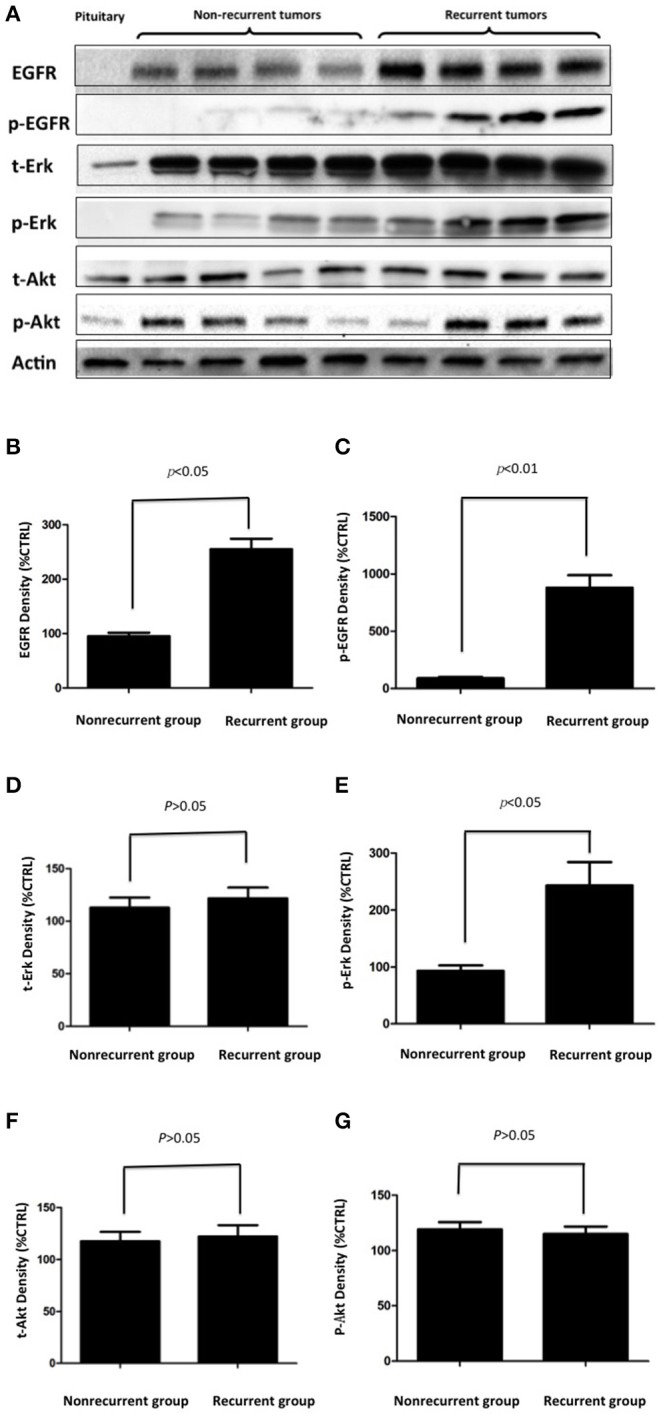
**(A)** The expression of EGFR and its signal transducing molecules p-EGFR, p-Akt, and p-Erk in EGFR-positive and EGFR-negative pituitary corticotroph adenomas and normal pituitary glands was measured by Western blot analysis. **(B–G)** Densitometric analysis of EGFR, p-EGFR, t-Erk, p-Erk, t-Akt, and p-Akt expression in recurrent and non-recurrent adenomas from the Western blot.

To validate EGFR expression in pituitary corticotroph adenomas and normal pituitary glands, Western blot analysis was used and showed a 170 kDa band for EGFR. The expression of EGFR was strong in corticotroph adenomas but not in normal pituitary glands (Figure 2A). Moreover, densitometric analysis revealed that the EGFR levels in the recurrent corticotroph adenomas were significantly increased compared to those in the non-recurrent corticotroph adenomas (Figure 2B).

To investigate EGFR downstream signaling, the EGFR signal transducing molecules phosphorylated EGFR (p-EGFR), total Akt, phosphorylated Akt (p-Akt), total Erk and phosphorylated Erk (p-Erk) were also investigated. p-EGFR and p-Erk were upregulated in recurrent adenomas but were not upregulated in non-recurrent adenomas or in normal pituitary glands, while the total Erk, total Akt, and p-Akt levels were unchanged (Figure 2A). Densitometric analysis showed significantly greater increases in p-EGFR and p-Erk levels in the recurrent adenomas than in the non-recurrent adenomas (all *p* < 0.05, Figures 2B–G).

### Association Between EGFR and the Clinicopathological Characteristics of Pituitary Corticotroph Adenomas

The relationships between the EGFR protein and the clinicopathological characteristics of corticotroph adenomas are described in Table 1. Using the Mann-Whitney test, the EGFR protein was found to be significantly associated with the recurrence status (*p* = 0.005), cortisol level (*p* = 0.009) and ACTH level (*p* = 0.008) but was not related with the sex, age, or symptom duration of the patient (*p* = 0.280, *p* = 0.351 and *p* = 0.142, respectively).

**Table 1:** Association between EGFR and the clinical characteristics of pituitary corticotroph adenomas.

	**Sex**	**Age**	**Symptom duration**	**Cortisol level**	**ACTH level**	**Recurrence interval**
		35.2 ± 12.4	4.6 ± 2.6	42.8 ± 16.5	82.8 ± 58.1	3.3 ± 2.1
Correlation coefficients	−0.204	−0.178	0.301	−0.446	−0.528	−0.488
*p-*value		0.351	0.142	0.009	0.008	0.005

## Discussion

CD is a rare disease, which has made informative studies underlying the adenoma recurrence with large cases difficult to perform. Moreover, due to the unavailability of human pituitary adenoma cell lines and reproducible animal models, the pace of translational research underlying the pathogenesis and progression was very slow until several studies showed that USP8, USP48, and BRAF are frequently mutated in pituitary corticotroph adenomas, causing CD by enhancing the promoter activity and transcription of the gene encoding POMC, which is the precursor of ACTH (8–10). Moreover, studies have shown that pituitary adenomas with mutated USP8 display an increased incidence of EGFR expression, EGFR protein abundance and the mRNA expression levels of POMC, indicating that EGFR plays an important role in adenomaigenesis (8, 9).

EGFR, a subtype of the ErbB receptors, which include EGFR (ErbB1, HER1), p185her2/neu (ErbB2, HER2), ErbB3 (HER3), and ErbB4 (HER4), can regulate cell motility and adhesion, adenoma invasion, angiogenesis, and adenoma cell proliferation (11). Receptor-specific ligands, including epidermal growth factor (EGF), heparin-binding EGF and transforming growth factor-α bind extracellular domains of individual ErbB receptors, cause receptor homo- or heterodimerization, and activate downstream signaling (12); this cascade results in adenoma initiation and progression. EGFR is expressed in normal anterior pituitary cells, including corticotrophs (13–17). Upregulating EGFR signaling leads to ACTH overproduction and adenoma growth in corticotroph adenomas (6). Although many studies have focused on the expression and function of EGFR in pituitary corticotroph adenomas, the association of EGFR with adenoma behavior, particularly the recurrence of pituitary corticotroph adenomas, was largely unknown. Here, we investigated the expression profile of EGFR and its pathway signaling in pituitary corticotroph adenomas and its relationship to adenoma clinicopathological characteristics.

In the present study, EGFR was overexpressed in 55.8% of the pituitary corticotroph adenomas and in 1 of 6 normal adenohypophysial tissues. The expression degree in pituitary corticotroph adenomas was significantly higher in pituitary corticotroph adenomas than in normal adenohypophysial tissues. Moreover, the EGFR signal transducing molecules p-EGFR, p-Akt and p-Erk were upregulated in EGFR-overexpressing adenomas but not in EGFR-negative adenomas or normal pituitary glands, indicating that EGFR and its downstream signaling pathway may be involved in adenomaigenesis.

Interestingly, the expression of EGFR was significantly higher in recurrent adenomas than in non-recurrent adenomas, indicating that EGFR may be considered a potential biomarker to determine and predict the recurrence likelihood of pituitary corticotroph adenomas. Although the possible reason of the significant association between EGFR overexpression and tumor recurrence needs to be investigated in more detail, the constitutive activation of EGFR by gene mutations and/or amplification has been shown to be related to initiation, progression, and poor prognosis in several cancers, including non-small cell lung cancer (NSCLC), colorectal cancer (CRC), squamous cell carcinoma of the head and neck (SCCHN), and glioblastoma (18, 19). The pituitary corticotroph adenomas with higher EGFR levels after surgery should be given more attention within a short follow-up period. Although there is no evidences showing EGFR overexpression and corticotroph tumor aggressiveness till now, EGFR overexpression predicted tumor progression and poor prognosis in breast cancer and prostate Cancer (20, 21).

Moreover, the expression levels of EGFR were positively correlated with ACTH and cortisol levels but not with age, sex, or adenoma size. The expression levels of EGFR were significantly increased in the recurrent group compared with those in the non-recurrent group (*p* < 0.01), and the expression levels were strongly related to the recurrence-free interval (*p* = 0.005, CC = −0.31). As EGFR is a potential therapeutic target of Cushing disease, two clinical trials including ours were initiated. In the clinical trial of Targeted Therapy With Gefitinib in Patients With USP8-mutated Cushing's Disease hosted by Huashan Hospital, China (NCT02484755), the researchers are still recruiting patients. And another clinical trial (ChiCTR-OPC-17011664) of clinical study of EGFR/HER2 targeted inhibitor Lapatinib in the treatment of refractory Cushing's disease initiated by our team are also recruiting patients.

As neither the late-night salivary cortisol test nor the desmopressin stimulation test was available in PUMCH, the Dexamethasone Test (ODT) and 24 h urinary free cortisol (UFC) may overlook patients with early recurrence. Now we are working with Clinical Laboratory Department try to initiate the late-night salivary cortisol test to identify the early recurrence cases.

## Conclusion

In our study, EGFR and its pathway signaling molecules had a higher expression in pituitary corticotroph adenomas than in normal pituitary glands. The EGFR levels were significantly correlated with recurrence status and a short disease-free interval. For those EGFR-overexpressing patients, more attention and intensive clinical and radiological follow-up may be needed after surgery. Although further confirmatory studies and more cases are needed, EGFR could be used as a promising biomarker for predicting pituitary corticotroph adenoma recurrence.

## Data Availability Statement

The raw data supporting the conclusions of this manuscript will be made available by the authors, without undue reservation, to any qualified researcher.

## Ethics Statement

The studies involving human participants were reviewed and approved by the Research Ethics Committee of Peking Union Medical College (Beijing, China). The patients/participants provided their written informed consent to participate in this study.

## Author Contributions

All authors listed have made a substantial, direct and intellectual contribution to the work, and approved it for publication.

### Conflict of Interest

The authors declare that the research was conducted in the absence of any commercial or financial relationships that could be construed as a potential conflict of interest.

## Abbreviations

ACTH: adrenocorticotropic hormone
CD: Cushing disease
EGFR: epidermal growth factor receptor
ERK: extracellularly regulated kinase
IOD: integrated optical density
POMC: proopiomelanocortin
USP8: ubiquitin specific peptidase 8.
